# Impact of the COVID-19 pandemic on lung cancer screening utilization and outcome: a health examination center retrospective study

**DOI:** 10.1080/07853890.2025.2525398

**Published:** 2025-06-28

**Authors:** Chi-Shen Chen, Cai-Sin Yao, Fu-Zong Wu

**Affiliations:** ^a^ Health Management Center, Kaohsiung Veterans General Hospital, Kaohsiung, Taiwan; ^b^ Department of Pharmacy, Chia Nan University of Pharmacy and Science, Taiwan, R.O.C; ^c^ Department of Business Management, National Sun Yat-Sen University, Kaohsiung, Taiwan; ^d^ Department of Medical Education and Research, Kaohsiung Veterans General Hospital, Kaohsiung, Taiwan; ^e^ Department of Radiology, Kaohsiung Veterans General Hospital, Kaohsiung, Taiwan; ^f^ Institute of Education, National Sun Yat-sen University, Kaohsiung, Taiwan; ^g^ Faculty of Medicine, School of Medicine, National Yang Ming Chiao Tung University, Taipei, Taiwan; ^h^ Faculty of Clinical Medicine, National Yang Ming Chiao Tung University, Taipei, Taiwan

**Keywords:** COVID-19, lung cancer screening, pandemic

## Abstract

**Background:**

The coronavirus disease 2019 (COVID-19) pandemic has disrupted healthcare systems, significantly affecting preventive services such as low-dose computed tomography (LDCT) for lung cancer screening. We aimed to evaluate the pandemic’s impact on LDCT screening practices at Kaohsiung Veterans General Hospital, focusing on the changes in participation rates, Lung-RADS categories, and lung cancer diagnoses to guide the development of interventions for improving screening programs and early detection during health crises.

**Materials and methods:**

A retrospective cohort of 56,730 individuals who underwent health examinations between 2017 and 2023 was analyzed. Data on demographics, smoking history, family history of lung cancer, and eligibility for LDCT subsidies in Taiwan were obtained. Screening utilization and outcomes were cross-referenced with cancer registries and imaging databases. A subset of 17,743 individuals who underwent LDCT were examined to determine the pre- and post-COVID19 differences in smoking prevalence, family history, high-risk Lung Imaging Reporting and Data System (Lung-RADS) categories (3 or 4), and lung cancer diagnoses.

**Results:**

Following the implementation of the level 3 alert, notable shifts were observed in smoking habits and lung cancer screening eligibility. The prevalence of heavy smokers (≥30 pack-years) declined from 6.9% before the alert to 6.1% after (*p* = 0.002). Conversely, the proportion of individuals with a family history of lung cancer qualifying for LDCT screening increased significantly from 6.0% to 6.6% (*p* = 0.009). Additionally, the prevalence of lung cancer diagnoses among individuals with high-risk Lung-RADS categories (Lung-RADS 3 or 4) decreased significantly from 21.5% before the alert to 13.4% after (*p* = 0.037).

**Conclusion:**

The pandemic disrupted LDCT screening, reducing access for high-risk smokers while increasing non-smoker participation. High-risk nodules declined but partially recovered post-pandemic. Future policies must prioritize high-risk individuals, optimize resources, and enhance early detection to improve outcomes and crisis preparedness.

## Introduction

The coronavirus disease 2019 (COVID-19) pandemic has led to unprecedented public health measures worldwide, with varying levels of success in controlling the spread of the virus [1]. In Taiwan, the highest level of alert (level 3) was implemented to mitigate the risk of COVID-19 transmission and protect public health [2,3]. We aimed to examine the impact and effectiveness of Taiwan’s level 3 alert measures, first instituted on 19 May 2020, and subsequently adjusted in response to the evolving pandemic situation.

During the level 3 alert, Taiwan implemented a series of stringent control measures to curb the spread of the virus [4]. These measures included restrictions on public gatherings, leading to the closure of schools, restaurants, and entertainment venues. Additionally, the government mandated wearing masks and maintaining social distancing in public spaces [5]. Border controls were intensified, imposing strict limitations on incoming travelers to prevent the introduction of new infections into the country. Furthermore, the scope of nucleic acid testing has been expanded, and vaccination efforts to boost population immunity have accelerated [6]. These comprehensive measures have been proven effective in managing the COVID-19 outbreak in Taiwan. As of 26 May 2023, Taiwan has reported a cumulative total of 15,300 confirmed cases and 807 deaths.

Lung cancer is the leading cause of cancer-related deaths worldwide and is consistently recognized by the World Health Organization (WHO) as the top contributor to cancer mortality [7]. Despite a 9-year decline in Taiwan’s standardized mortality rate for lung cancer, the disease accounted for 9629 deaths in 2020, constituting approximately 19.2% of total cancer fatalities [8]. The prognosis of patients with lung cancer is strongly influenced by the stage at diagnosis, with significant disparities in the 5-year survival rates between early-stage and late-stage diagnoses. For example, patients diagnosed with stage 1 disease exhibit an overall survival rate of approximately 90%, whereas those with stage IV disease have a survival rate of only 10% [9].

The COVID-19 pandemic has significantly impacted healthcare systems, leading to the reallocation of resources. Previous studies have examined the operational challenges of LCS during the pandemic, but there is limited research on LDCT screening behavior and eligibility criteria specific to Asian populations [10]. This study aimed to explore the impact of the COVID-19 pandemic on lung cancer screening practices at health examination centers, with a particular focus on the use of low-dose computed tomography (LDCT) for lung cancer screening, contributing to a better understanding of screening behavior and potential adaptations for future public health crises. The pandemic may have had a profound impact on an individual’s health awareness and behavioral patterns, particularly in relation to health checkups and screening [11–13]. The outbreak may have altered individuals’ perceptions of health risks, heightened awareness of the importance of screening, and affected their participation in screening programs [14,15]. Therefore, the primary goal of this study was to assess whether significant changes occurred in LDCT lung cancer screening behavior during the pandemic, investigate the factors driving these changes, and examine the subsequent distribution of Lung-RADS lung nodule categories and lung cancer diagnoses. The findings of this study will aid in the development of targeted interventions to improve lung cancer screening and diagnosis during this global health crisis.

## Methods

**Figure 1.**
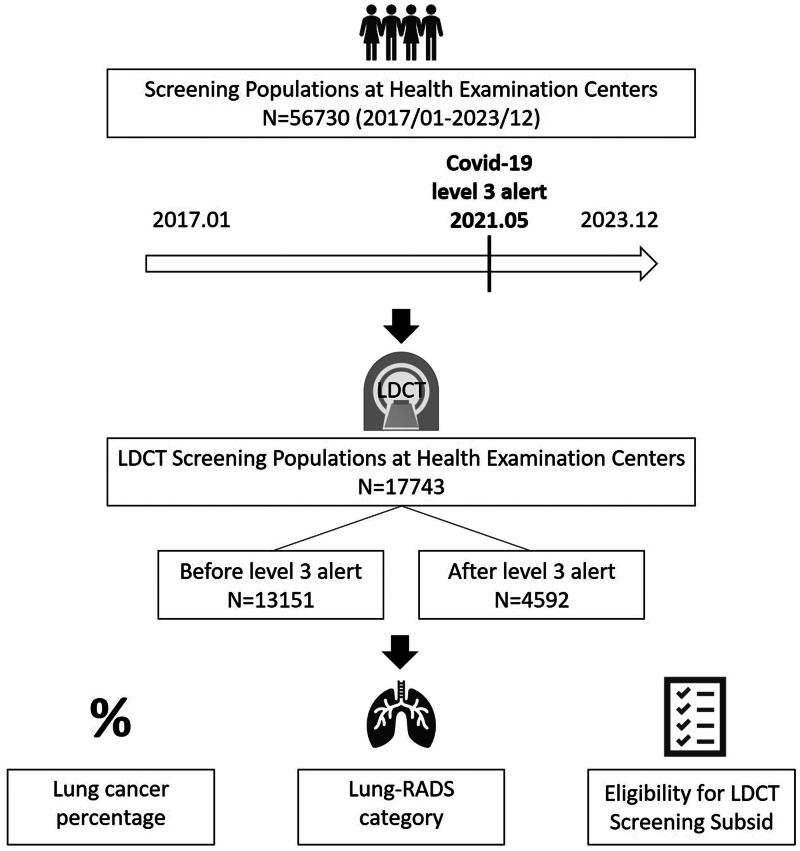
Study design and flowchart.

We retrospectively collected data from all individuals (*N* = 56,730) who underwent health examinations at the Health Screening Center of Kaohsiung Veterans General Hospital between January 2017 and December 2023. The study flowchart is shown in Figure 1. Figure 1 includes the complete cohort of individuals screened at health examination centers (*N* = 56,730), regardless of the screening method used. In contrast, it focuses on the subgroup that underwent LDCT screening, representing *N* = 17,743 participants from the overall population. Table 1 includes the full group of individuals screened at health examination centers (*N* = 56,730), irrespective of the screening method used. In contrast, Table 2 specifically highlights the subgroup that underwent LDCT screening, comprising *N* = 17,743 participants from the overall population. This retrospective analysis was approved by the Institutional Review Board (IRB) of Kaohsiung Veterans General Hospital (IRB: KSVGH24-CT3-5). This study adheres to the principles outlined in the Declaration of Helsinki, ensuring the ethical conduct of research involving human participants. Due to the retrospective design of the study, the board waived the requirement for written informed consent. The participants were categorized based on LDCT lung screening status. Baseline demographic information including age, sex, smoking habits, and the region of residence were obtained from the electronic medical records. Health questionnaires were used to collect additional data, such as smoking history, symptoms of chest discomfort, a history of pulmonary disease, and a family history of lung cancer. The participants were further classified based on Taiwan’s LDCT lung cancer screening subsidy eligibility criteria, which include heavy smokers (30 pack-years), individuals with a family history of lung cancer within three degrees of kinship, and individuals meeting both criteria. Additionally, the collected data were cross-referenced with cancer registries and imaging databases to determine the proportion of individuals who underwent LDCT screening and the overall proportion of patients diagnosed with lung cancer. Between January 2017 and December 2023, 17,743 individuals who underwent LDCT were specifically analyzed. The study also compared the differences in smoking prevalence, family history of lung cancer, eligibility for LDCT screening based on smoking or family history, the proportion of high-risk Lung-RADS nodules (Lung-RADS categories 3 or 4), and lung cancer diagnoses in the pre- and post-COVID periods (Taiwan’s level 3 alert measures) [16]. This retrospective cohort study aimed to evaluate the influence of level 3 alert measures on LDCT screening uptake and lung cancer detection rates among individuals who underwent health examinations at the Kaohsiung Veterans General Hospital between 2012 and 2023.

**Table 1.** Clinical characteristics of screening populations at health examination centers before and after the COVID-19 level 3 alert.

Variables	Total *n* = 56,730(%)	Before level 3 alert *n* = 42,583 (75.1%)	After level 3 alert *n* = 14,147(24.9%)	*p*-Value
Age (mean±*SD*), year	49.2 ± 11.4	49.1 ± 11.4	49.6 ± 11.1	<0.001
Age				0.072
≤55 y/o	40,528 (71.4)	30,505 (71.6)	10,023 (70.8)	
>55 y/o	16,202 (25.6)	12,078 (28.4)	4124 (29.2)	
Age				<0.000
<50 y/o	28,682 (50.6)	21,891 (51.4)	6791 (48)	
≧50 y/o	28,048 (49.4)	20,692 (48.6)	7356 (52)	
Sex				0.046
Male	29,448 (51.9)	22,207 (52.2)	7241 (51.2)	
Female	27,282 (48.1)	20,376 (47.8)	6906 (48.8)	
Smoking habit				0.002
≥30 pack year	3800 (6.7)	2934 (6.9)	866 (6.1)	
<30 pack year	52,930 (93.3)	39,649 (93.1)	13,281 (93.9)	
Residential location				<0.001
South Taiwan	38,053 (90.9)	28,795 (90.0)	9258 (93.8)	
Not South Taiwan	3789 (9.1)	3181 (10.0)	608 (6.2)	
Smoking habit				0.002
≥30 pack year	3800 (6.7)	2934 (6.9)	866 (6.1)	
<30 pack year	52,930 (93.3)	39,649 (93.1)	13,281 (93.9)	
Chest symptom	6527 (11.5)	5133 (12.1)	1394 (9.9)	<0.001
History about respiratory symptoms	11,766 (20.7)	9377 (22.0)	2389 (16.9)	<0.001
History of respiratory disease	2582 (4.6)	2079 (4.9)	503 (3.6)	<0.001
Family history of lung cancer	5722 (10.1)	2079 (4.9)	503 (3.6)	<0.001
**Eligibility for LDCT Screening Subsidy**				
Heavy smokers	2308 (4.1)	1753 (4.1)	555 (3.9)	0.313
Family history of lung cancer eligible for LDCT screening	3478 (6.1)	2546 (6.0)	932 (6.6)	0.009
Both	5530 (9.8)	4106 (9.6)	1424 (10.1)	0.141
LDCT	17,743 (31.3)	13,151 (30.9)	4592 (32.5)	<0.001
Lung cancer	432 (0.8)	334 (0.8)	98 (0.7)	0.277

Respiratory disease: Asthma, Tuberculosis, Chronic bronchitis, Pulmonary emphysema, Chronic Obstructive Pulmonary Disease.

Respiratory symptom: Cough/Dyspnea/Breathless when exercising.

Chest symptom: Chest pain.

**Table 2.** Clinical characteristics of LDCT screening populations at health examination centers before and after the COVID-19 level 3 alert.

Variables	Total *n* = 17,743(%)	Before level 3 alert *n* = 13,151 (74.1%)	After level 3 alert *n* = 4592(25.9%)	*p*-Value
Age (mean ± SD), year	53.5 ± 11.0	53.4 ± 11.0	53.6 ± 11.1	0.519
Age				0.876
≤55 y/o	10,048 (56.6)	7443 (56.6)	2605 (56.7)	
>55 y/o	7695 (43.4)	5708 (43.4)	1987 (43.3)	
Age				0.287
<50 y/o	6328 (35.7)	4720 (35.9)	1608 (35.0)	
≧50 y/o	11,415 (64.3)	8431 (64.1)	2984 (65.0)	
Sex				0.008
Male	10,412 (58.7)	7793 (59.3)	2619 (57.0)	
Female	7331 (41.3)	5358 (40.7)	1973 (43.0)	
Residential location				<0.001
South Taiwan	13,147 (87.7)	9629 (86.5)	3518 (91.2)	
Not South Taiwan	1843 (12.3)	1505 (13.5)	338 (8.8)	
Lung-RADS				<0.001
Lung-RADS 1	12,480 (70.3)	9722 (73.9)	2758 (60.1)	
Lung-RADS 2	4754 (26.8)	3062 (23.3)	1692 (36.8)	
Lung-RADS 3	252 (1.4)	183 (1.4)	69 (1.5)	
Lung-RADS 4	257 (1.5)	184 (1.4)	73 (1.6)	
Lung-RADS risk				0.292
Low risk	17,234 (97.1)	12,784 (97.2)	4450 (96.9)	
High risk	509 (2.9)	367 (2.8)	142 (3.1)	
Smoking habit				<0.001
≥30 pack year	1992 (11.2)	1544 (11.7)	448 (9.8)	
<30 pack year	15,751 (88.8)	11,607 (88.3)	4144 (90.2)	
Chest symptom	3059 (17.2)	2415 (18.4)	644 (14.0)	<0.001
History about respiratory symptoms	4875 (27.5)	3866 (29.4)	1009 (22.0)	<0.001
History of respiratory disease	1048 (5.9)	844 (6.4)	204 (4.4)	<0.001
Family history of lung cancer	2186 (12.3)	1585 (12.1)	601 (13.1)	0.066
*Eligibility for LDCT Screening Subsidy*				
Heavy smokers	1286 (7.2)	995 (7.6)	291 (6.3)	0.006
Family history of lung cancer Eligible for LDCT screening	1487 (8.4)	1078 (8.2)	409 (8.9)	0.135
Both	2636 (14.9)	1966 (15)	670 (14.6)	0.556
Lung cancer	221 (1.3)	171 (1.3)	50 (1.1)	0.266

### Statistical analysis

Descriptive statistics were used to summarize the characteristics of the study population. Chi-square and *t*-tests were used to compare categorical and continuous variables between the groups, respectively. In addition, utilize trend plots over the years to visually present changes and support temporal analysis.

## Results

### General health checkup cohort

The study population comprised 56,730 individuals who underwent health examinations between 2017 and 2022. Of these, 42,583 individuals (75.1%) underwent examinations before the implementation of level 3 alert, whereas 14,147 individuals (24.9%) underwent examinations after the implementation of the alert (Table 1). The average age of individuals was slightly higher after the alert (49.6 ± 11.1 years) compared with that before the alert (49.1 ± 11.4 years), showing a significant difference (*p* < 0.001). When categorized by age, the proportion of individuals aged ≥50 years increased significantly after the alert, from 48.6% to 52% (*p* < 0.001). Additionally, the gender distribution slightly changed but significantly, with the proportion of women increasing from 47.8% to 48.8% after the alert (*p* = 0.046).

Smoking habits demonstrated a notable shift, with the prevalence of heavy smokers (≥30 pack years) decreasing from 6.9% before the alert to 6.1% after the alert (*p* = 0.002). The overall distribution of smoking habits also demonstrated significant differences before and after the alert (*p* = 0.002). A geographical shift was observed, with a significant increase in the proportion of individuals from South Taiwan after the alert (from 90.0% to 93.8%) (*p* < 0.001). In terms of chest symptoms and disease history, a significant decrease was noted in the proportion of individuals reporting chest symptoms (from 12.1% before the alert to 9.9% after the alert) (*p* < 0.001). Similarly, the proportion of individuals who developed respiratory symptoms decreased from 22.0% to 16.9% (*p* < 0.001), and the proportion of individuals with a history of lung disease decreased from 4.9% to 3.6% (*p* < 0.001). Conversely, the proportion of individuals with a family history of lung cancer increased significantly after the alert (from 4.9% to 6.6%) (*p* < 0.001).

The implementation of the level 3 alert had a significant impact on LDCT screening and lung cancer detection rates. The proportion of individuals who underwent LDCT increased from 30.9% before the alert to 32.5% after the alert (*p* < 0.001). However, the proportion of heavy smokers eligible for LDCT screening remained stable at 3.9% before the alert and 4.1% after the alert (*p* = 0.313). By contrast, the proportion of individuals with a family history of lung cancer eligible for LDCT screening significantly increased from 6.0% to 6.6% (*p* = 0.009). The proportion of individuals eligible for both criteria remained unchanged at 10.1% before the alert and 9.6% after the alert (*p* = 0.141). Despite these changes, the overall lung cancer detection rate did not show a significant difference before and after the alert, remaining at 0.7% and 0.8%, respectively, in the general health checkup population (*p* = 0.277).

### Healthy checkup LDCT cohort

The baseline characteristics of individuals who underwent LDCT screening are presented in Table 2. The study population consisted of 17,743 individuals, with 13,151 (74.1%) undergoing LDCT before the level 3 alert and 4592 (25.9%) after the alert. The average age of individuals who underwent LDCT screening remained consistent before and after the alert (53.4 ± 11.0 years and 53.6 ± 11.1 years, respectively; *p* = 0.519). When categorized by age, no significant difference was observed between individuals aged <50 years and those aged ≥50 years (65.0% vs. 64.1%; *p* = 0.287). However, the proportion of women who underwent LDCT screening significantly increased after the alert (from 40.7% to 43.0%) (*p* = 0.008). Additionally, the proportion of individuals from South Taiwan who underwent LDCT screening significantly increased after the alert (from 86.5% to 91.2%) (*p* < 0.001).

**Figure 2.**
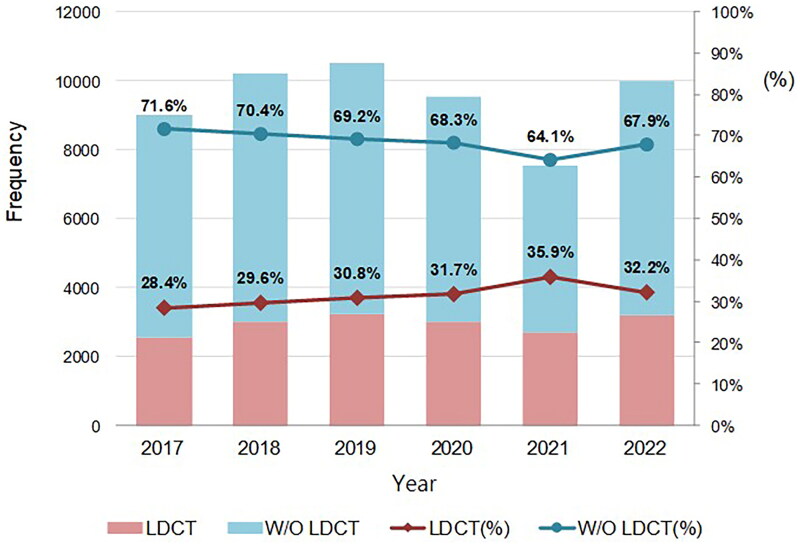
Comparison of individuals Undergoing LDCT and those without LDCT in health checkups, 2017–2022.

The distribution of Lung-RADS categories also showed significant changes after the alert. The proportion of individuals classified as Lung-RADS 1 decreased from 73.9% before the alert to 60.1% after the alert, whereas that classified as Lung-RADS 2 increased from 23.3% to 36.8% (*p* < 0.001). The proportions of individuals classified as Lung-RADS 3 and 4 remained relatively stable (1.5% vs. 1.4% and 1.6% vs. 1.4%, respectively). In terms of smoking habits, the proportion of heavy smokers (≥30 pack years) who underwent LDCT screening decreased significantly from 11.7% before the alert to 9.8% after the alert (*p* < 0.001). The overall smoking habits also showed significant differences before and after the alert (*p* < 0.001). The proportion of individuals reporting chest symptoms decreased significantly after the alert (from 18.4% to 14.0%) (*p* < 0.001). Similarly, the proportion of individuals with a history of respiratory symptoms decreased from 29.4% to 22.0% (*p* < 0.001), while the proportion of those with lung diseases decreased from 6.4% to 4.4% (*p* < 0.001). The proportion of individuals with a family history of lung cancer showed a slight, non-significant increase after the alert (from 12.1% to 13.1%) (*p* = 0.066). The bar and line chart (Figure 2) shows a comparison between individuals who underwent LDCT and those who did not (W/O LDCT) during health checkups from 2017 to 2022. Each bar represents the total number of participants per year, divided between those who underwent LDCT screening (red) and those who did not (blue). The chart also displays the proportion of individuals within each category, with the LDCT screening rate indicated by red diamonds and the non-LDCT screening rate indicated by blue circles. Notably, the LDCT rate increased from 28.4% in 2017 to 32.2% in 2022, whereas the non-LDCT screening rate decreased from 71.6% to 67.9%, indicating a gradual shift toward increased LDCT usage in health checkups over time.

**Figure 3.**
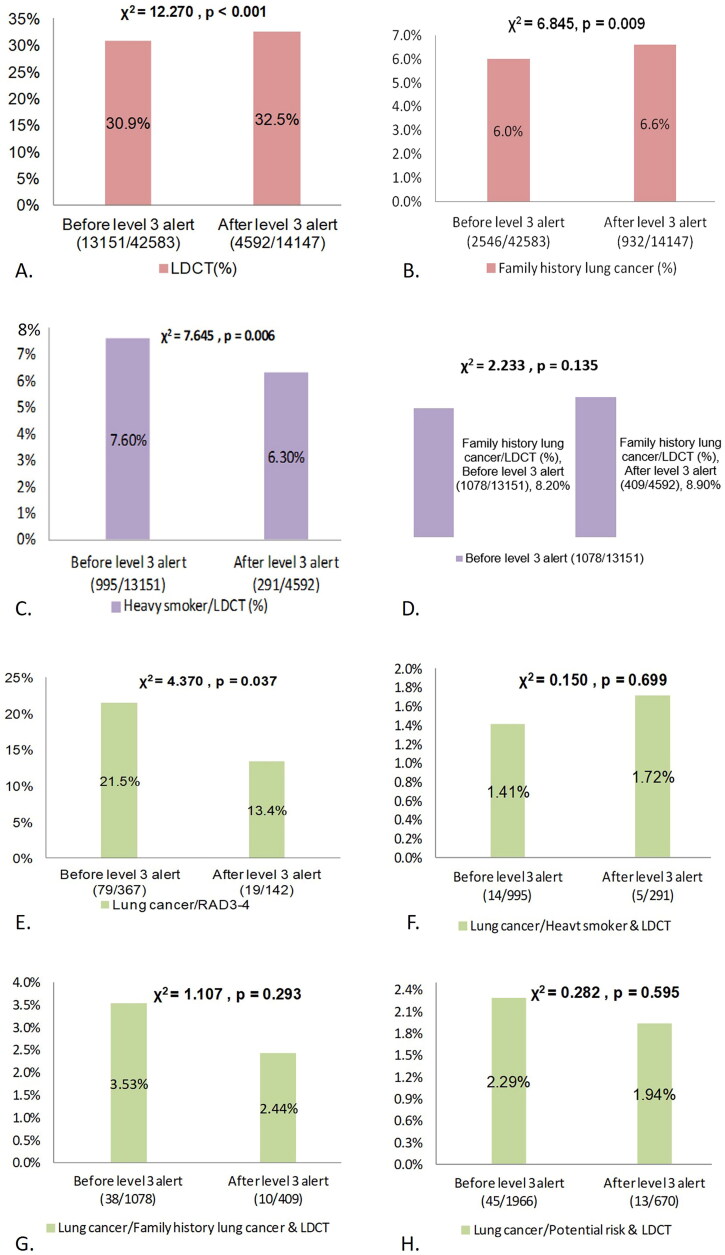
(A) Proportion of individuals Undergoing LDCT before and after level 3 alert. (B) Proportion of individuals with family history of lung cancer (eligible for LDCT subsidy) before and after level 3 alert. (C) Proportion of heavy smokers (eligible for LDCT subsidy) among LDCT recipients before and after level 3 alert. (D) Proportion of individuals with family history of lung cancer (eligible for LDCT subsidy) among LDCT recipients before and after level 3 alert. (E) Proportion of lung cancer patients with RAD3-4 scores in LDCT screening before and after level 3 alert. (F) Proportion of lung cancer patients among heavy smokers Undergoing LDCT screening before and after level 3 alert. (G) Proportion of lung cancer patients with a family history of lung cancer among LDCT screening cases before and after level 3 alert. (H) Proportion of lung cancer patients among individuals with potential lung cancer risk factors Undergoing LDCT screening before and after level 3 alert.

Annual analysis of LDCT screening before and after the implementation of the level 3 alert (Figure 3(A)) revealed a significant increase in the proportion of individuals who underwent LDCT screening. Before the alert, 13,151 individuals (30.9%) underwent LDCT screening; after the alert, only 4592 individuals (32.5%) underwent LDCT screening. Non-LDCT screening accounted for 69.1% of examinations before the alert and 67.5% after the alert. These data indicate that the implementation of the level 3 alert may have positively influenced the uptake of LDCT screening for lung cancer detection.

The impact of level 3 alert on individuals with a family history of lung cancer is illustrated in Figure 3(B). The proportion of individuals with a family history of lung cancer increased significantly from 6.0% before the alert to 6.6% after the alert (*p* = 0.009). This suggests that the implementation of the level 3 alert may have encouraged more individuals with a family history of lung cancer to participate in health examinations and potentially in LDCT screening.

By contrast, the implementation of the level 3 alert had a negative impact on the participation of heavy smokers in LDCT screening (Figure 3(C)). The proportion of heavy smokers who underwent LDCT screening decreased significantly from 7.60% before the alert to 6.30% after the alert (*p* = 0.006). The proportion the individuals with a family history of lung cancer who underwent LDCT screening (Figure 3(D)) increased slightly, but was not significant (from 8.20% before the alert to 8.90% after the alert) (*p* = 0.066).

The prevalence of lung cancer diagnosis in patients with high-risk Lung-RADS categories (Lung-RADS 3 or 4) was also affected by the implementation of the level 3 alert (Figure 3(E)). The proportion of individuals in these high-risk categories who were diagnosed with lung cancer significantly decreased from 21.5% before the alert to 13.4% after the alert (*p* = 0.037). This decrease suggests that the implementation of the level 3 alert may have influenced the lung cancer diagnosis rates among high-risk individuals. However, no significant difference was found in the lung cancer detection rates among heavy smokers who underwent LDCT screening before and after the alert (Figure 3(F)). The detection rate slightly increased from 1.41% to 1.72%; however, this change was not significant (*p* = 0.699).

The analysis of lung cancer diagnoses among individuals with a family history of lung cancer who underwent LDCT screening before and after the implementation of the level 3 alert (Figure 3(G)) showed a slight decrease in the rate of diagnoses, from 3.53% before the alert to 2.44% after the alert. However, this difference was not significant (*p* = 0.29). Similarly, the analysis of lung cancer diagnosis rate among individuals with potential risks who underwent LDCT screening (Figure 3(H)) demonstrated a slight decrease in the diagnosis rate from 2.29% before the alert to 1.94% after the alert; however, this change was not significant (*p* = 0.59).

**Figure 4.**
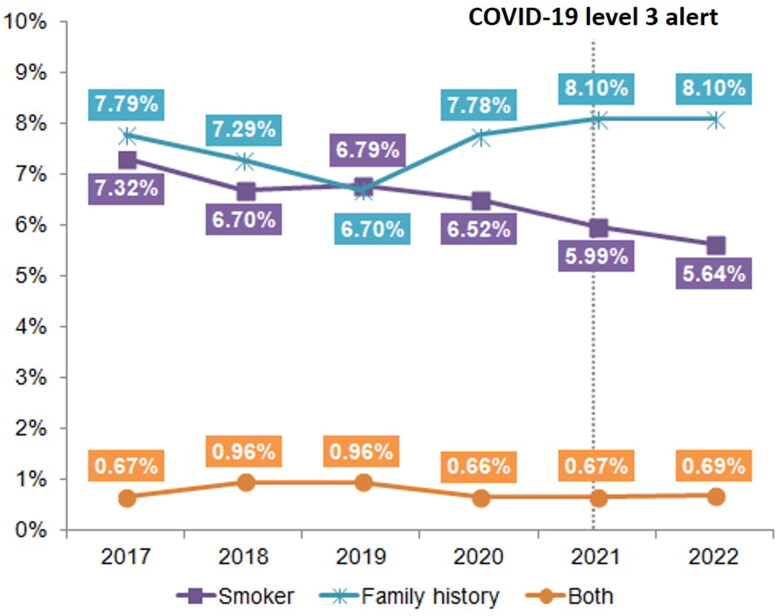
Proportion of individuals meeting eligibility criteria for LDCT screening subsidy (smoker, family history, or both) from 2017 to 2022.

The annual analysis of smoking habits and family history of lung cancer from 2017 to 2022 (Figure 4) showed a decreasing trend in the proportion of smokers in the LDCT screening cohort from 7.32% in 2017 to 5.64% in 2022. By contrast, the proportion of individuals with a family history of lung cancer slightly fluctuated but showed an overall increase from 7.79% in 2017 to 8.10% in 2022. The proportion of individuals with smoking habits and a family history of lung cancer remained consistently low, ranging from 0.66% to 0.96%. This analysis indicated a shift in the demographics of individuals undergoing LDCT screening, with a reduction in the proportion of smokers and a stable proportion of individuals with a family history of lung cancer. These trends may reflect the impact of the implementation of public health initiatives and awareness campaigns focused on smoking cessation and the importance of lung cancer screening, particularly in individuals with a family history of the disease.

## Discussion

Cancer screening is an essential component of cancer prevention and control strategies [17]. It aims to detect cancer at an early stage, when treatment is most effective, thereby reducing cancer mortality and improving patient outcomes. However, disparities in access to and utilization of cancer screening persist, leading to unequal cancer burdens and outcomes across different population groups [18–21]. The COVID-19 pandemic has further exacerbated these disparities, posing significant challenges for cancer prevention efforts [22–24]. Studies have consistently reported a substantial decline in screening rates for various cancer types, including breast, colorectal, and cervical cancers. This reduction was observed across different age groups, ethnicities, and socioeconomic backgrounds [25,26]. The decline in screening rates is particularly pronounced among vulnerable populations such as racial and ethnic minorities, individuals with low socioeconomic status, and those living in rural areas [11,27]. These disparities highlight the exacerbating effects of the pandemic on existing inequities in cancer screening access and utilization [28]. The decline in screening rates raises serious concerns about potential delays in cancer diagnosis, leading to an increase in cancer mortality rates [29]. Early cancer detection is crucial for improving treatment and survival outcomes; therefore, pandemic-related disruptions in screening could have long-term implications on cancer burden [30]. For example, breast cancer screening rates declined by 30–80% during the pandemic compared with pre-pandemic levels, with Black and Hispanic women experiencing a more significant decline compared with White women [31,32]. Similarly, colorectal cancer screening showed reductions ranging from 20% to 60%, especially in individuals with lower socioeconomic status and living in rural areas [33,34]. The cervical cancer screening rates were also significantly affected, with some studies reporting reductions of up to 90% [35,36]. This decline is particularly concerning given the critical role of cervical cancer screening in early detection and disease prevention.

Adherence to cancer screening and the impact of the COVID-19 pandemic on screening rates are key factors influencing the effectiveness of cancer screening [37]. The COVID-19 pandemic has significantly affected cancer screening programs, leading to a decline in screening rates across various cancer types, including breast, colorectal, and cervical cancers [11,38]. This reduction was observed across different demographics but was particularly pronounced among vulnerable populations, such as racial and ethnic minorities, individuals with low socioeconomic status, and those living in rural areas [24]. This decrease in screening rates raises concerns about potential delays in cancer diagnosis and increased long-term cancer mortality rates [39–41].

The relationship between smoking and lung cancer is well documented, with smoking being the primary risk factor for lung cancer development [42]. The efficacy of screening programs, particularly LDCT, in reducing lung cancer mortality has been a significant focus of recent research [43,44]. This discussion synthesizes the findings from various studies, including randomized controlled trials, to evaluate the current understanding and future directions of lung cancer screening [45]. Smoking accounts for approximately 85% of all lung cancer cases [46]. Carcinogens in tobacco smoke induce mutations in critical genes, leading to the malignant transformation of lung epithelial cells. The risk of lung cancer increases with the duration and intensity of smoking, with heavy smokers experiencing the highest risk. Despite smoking cessation efforts, former smokers remain at an elevated risk, necessitating effective screening strategies [47]. LDCT has emerged as the most effective screening modality for high-risk populations, particularly heavy smokers and former smokers. The National Lung Screening Trial demonstrated a 20% reduction in lung cancer mortality with LDCT screening compared with chest radiography [48]. Subsequent European studies, such as the NELSON trial, corroborated these findings, showing a 26% and 39% reduction in lung cancer mortality among men and women, respectively [44]. The increasing acceptance of LDCT for lung cancer screening among Asians is a notable trend, particularly post-COVID-19 pandemic [49]. Traditionally, conservative Asian societies have shown growing openness to health screening, especially after the COVID-19 outbreak. Therefore, the purpose of this study and the rationale for conducting it are to gain a deeper understanding of the pandemic’s impact and the real-world effectiveness of lung cancer screening in the Asian population.

This study highlighted four key findings. First, a significant surge in LDCT scan uptake occurred in Asia post-COVID-19, which is likely attributable to the heightened public awareness of respiratory health risks during the pandemic. Given the direct impact of COVID-19 on lung function, individuals have become more cognizant of their lung health and have shown more willingness to undergo screening for early disease detection [50]. Moreover, post-pandemic healthcare campaigns and the proliferation of digital health management have raised awareness of the value of LDCT screening, further driving its increased uptake [51].

Second, among those who underwent LDCT screening after the pandemic, an increasing proportion of patients undergoing lung cancer screening (LCS) had a family history of smoking, whereas a decreasing proportion had a prior history of smoking. This trend may reflect a shift in public health awareness. As the pandemic progressed, non-smokers began to recognize that lung cancer risks extended beyond smoking, especially for those with a family history. This shift may also be attributed to the rising incidence of lung cancer in non-smokers in Asia, prompting individuals with a family history to undergo LDCT screening out of concern [52]. These findings may reflect changes in health-seeking behaviors or shifting demographics of screening participants during the pandemic period, but we acknowledge that further studies are needed to confirm whether these patterns have meaningful implications for lung cancer risk or screening policy.

This study addresses the real-world Asian population, focusing on the non-smoking-related lung cancer burden and screening behaviors. Unlike NLST and NELSON criteria, which emphasize smoking history, Taiwan is the first country to expand eligibility, allowing high-risk individuals to apply for screening if they meet either of the following criteria: (1) Men (50–74 years) and women (45–74 years) with a first-degree relative diagnosed with lung cancer, or (2) Individuals (50–74 years) with *a* ≥ 30 pack-year smoking history, currently smoking or quit within 15 years [48]. Additionally, the pandemic may have encouraged some smokers to quit, thereby reducing the proportion of smokers among those screened. These changes indicate the diversification of the screening population, with an increasing demand for screening among non-smokers [53].

Third, the proportion of Lung-RADS category 3 and 4 high-risk lung nodules diagnosed as lung cancer decreased after the level 3 alert period. This may be due to several factors, including the reallocation of medical resources during the pandemic, which led to delays in screening for high-risk individuals or postponement of medical visits [54]. During the post-alert period, the proportion of Lung-RADS high-risk nodules diagnosed as lung cancer was significantly lower than that in the pre-alert period. This reduction may be attributed to a shortage in medical resources during the COVID-19 outbreak, limiting opportunities for patients to undergo further evaluation at hospitals. Therefore, special attention is needed in the post-COVID period to prevent delays in lung cancer diagnosis, as such delays could adversely affect patient survival rates [55].

Several factors may have contributed to this finding. These include the potential reallocation of healthcare resources during the pandemic, leading to delays in screening for high-risk individuals or treatment delays among patients. Additionally, the increased screening rate among non-smokers may have influenced the proportion of high-risk nodules detected. As LDCT screening expands beyond the traditional high-risk smoking population to include low-risk non-smokers, detection rates may fluctuate [56]. Fourth, the overall trend in lung cancer screening after post-COVID-19 indicates a broader adoption of LDCT, with a shift in the screening population from the traditional high-risk smoking population to non-smokers with a family history of lung cancer. As health consciousness improves and medical technology advances, early lung cancer screening will remain a vital preventive measure in Asia, with the risk distribution of the screening population expected to evolve [52]. Among individuals who underwent LDCT, the proportion of those with a family history of lung cancer gradually increased, whereas the proportion of smokers decreased [57]. This trend reflects a shift in health awareness and behavior.

The outbreak of COVID-19 significantly altered the attitudes and behaviors of the Taiwanese population toward lung cancer screening, thereby impacting the allocation of healthcare resources and policy responses. Owing to the threat of the pandemic, many individuals became hesitant to undergo lung cancer screening, especially during the early stages when hospitals were perceived as high-risk environments [58]. This behavioral shift not only affected people’s willingness to undergo screening but also influenced public health strategies and the utilization of healthcare resources. The outbreak prompted Taiwan’s healthcare system to reassess lung cancer screening policies, implement restrictions on nonurgent examinations to reduce hospital traffic, enhance safety protocols for healthcare workers and patients, and maintain healthcare stability with limited resources [59]. During the COVID-19 pandemic, some hospitals in Taiwan have adjusted their lung cancer screening policies, including the suspension or postponement of non-urgent screenings to minimize the risk of virus transmission. However, these policy changes have led to delays in screening for high-risk groups, thereby increasing the risk of delayed lung cancer diagnoses. As the pandemic gradually subsided, the surge in screening demand placed significant pressure on healthcare resources as individuals resumed seeking medical services. To address the anticipated increase in screening demand in the post-COVID era, healthcare systems have implemented measures such as establishing expedited screening channels and increasing budget allocations for screening services to accommodate the deferred demand.

Our findings demonstrated a significant difference in lung cancer screening (LCS) attendance between the pre-COVID and COVID years in the Asian population, highlighting the resilience and continuity of screening programs despite pandemic-related challenges. This is particularly relevant given the rising epidemic of lung cancer among non-smokers in Asia. In contrast, studies from Western populations reported no significant difference in LCS attendance between the pre-COVID and COVID periods, underscoring the need for region-specific strategies to optimize screening participation [60]. Future research should explore post-pandemic trends and outcomes in LCS attendance, considering the impact of fluctuating transmission rates and evolving healthcare accessibility. Additionally, real-world variations in screening behaviors among Asian populations should be examined, as differences in health literacy, cultural perceptions of lung cancer risk, and biological factors may influence screening uptake. A deeper understanding of these disparities can help develop more tailored and effective screening strategies, ensuring equitable access and improved adherence across diverse populations.

The pandemic has prompted Taiwan’s healthcare institutions to reconsider and refine lung cancer screening policies to adapt to post-pandemic needs. These adaptations include prioritizing access for high-risk groups, enhancing screening promotion in primary care settings, and integrating lung cancer screening into broader public health policies [61]. However, current research investigating the behavioral changes after the pandemic remains limited. For example, the lack of survey data prevents in-depth insights into the specific factors influencing screening behaviors during and after the pandemic. Further empirical analysis is needed to determine whether concerns about screening risks have increased or if expectations for screening results have shifted due to the influence of the pandemic.

Additionally, no survey studies have focused on assessing the policy responses of healthcare institutions during or after the pandemic. Surveying healthcare institutions regarding policy changes, such as implementing stricter screening controls during the pandemic or increasing resource allocation in the post-COVID phase to address the surge in screening demand, could provide a more comprehensive understanding of healthcare resource allocation and policy adjustments. Such studies could illuminate the impact of the pandemic on the healthcare system’s response to lung cancer screening and guide future resource allocation strategies to mitigate screening delays for high-risk populations in similar public health crises. a formal trend analysis to detect an inflection point was not feasible due to two key limitations. This study has two limitations. First, the data are cross-sectional and derived from independent annual health examination records, not a longitudinal cohort. This limits the ability to infer true temporal trends, as year-to-year differences may reflect variations in population composition. Second, multiple external factors beyond the pandemic—such as evolving health policies or awareness campaigns—could have influenced screening behaviors. Without the ability to control for these confounders, attributing observed changes solely to the Level 3 alert would be speculative.

Our study examines pre- and post-COVID-19 trends, real-world evidence, and the characteristics of the Asian population within the LDCT program, considering both smoking and non-smoking groups. While matched analysis was not feasible in this study due to certain limitations, including the lack of comparable baseline data and the heterogeneous nature of the study population, we plan to further explore these factors in future research. This will help strengthen our findings and minimize potential confounding variables, such as differences in healthcare access, socioeconomic status, and regional variations in screening practices. In summary, the COVID-19 pandemic has profoundly changed the behavior of Taiwanese people toward lung cancer screening and has prompted healthcare systems to reconsider and adjust their resource allocation and policies (62),(63). Future research should further explore the behavioral and policy changes triggered by the pandemic to enhance screening services and strengthen safeguards for lung cancer screening in similar public health challenges.

## Conclusion

During the pandemic, the reallocation of healthcare resources may lead to delays in lung cancer screening, particularly for high-risk Lung-RADS cases, as non-urgent services were limited. The reallocation of healthcare resources contributed to a decline in lung cancer diagnoses, particularly among high-risk Lung-RADS cases, likely due to fewer procedures being conducted. Additionally, this study did not investigate the effect outcome of delayed diagnoses or changes in cancer staging.

The early detection of potential lung cancer cases was affected; after the pandemic, screening demand shifted, with an increase in screening among non-smokers, likely due to increased health awareness. However, screening rates among high-risk heavy smokers have declined, raising concerns about missed diagnoses. During the post-pandemic period, healthcare systems must adjust screening policies to address the needs of different groups, especially heavy smokers, preventing delays in lung cancer detection. Optimizing resources to meet the growing demand among non-smokers through education and risk assessment is crucial. In summary, the pandemic-induced redistribution of healthcare resources had a profound impact on lung cancer screening behaviors and outcomes across different population groups. Future policies should consider the distinct screening needs of each group and develop more effective screening strategies to achieve early diagnosis and reduce lung cancer mortality.

## Supplementary Material

STROBE 20250225.doc

## Data Availability

Data supporting the findings of this study are available from the corresponding author, F. Z. W., upon reasonable request.
